# Robot-mediated impairment-oriented and task-specific training on upper limb post stroke: feasibility and preliminary effects on physical function and quality of life

**DOI:** 10.3389/fneur.2024.1415773

**Published:** 2024-10-11

**Authors:** San San Tay, Fuquan Zhang, Christine Alejandro Visperas, Xuan Han Koh, Borisut Lau, Jin Rui Edmund Neo

**Affiliations:** ^1^Department of Rehabilitation Medicine, Changi General Hospital, Singapore, Singapore; ^2^Department of Health Services Research, Changi General Hospital, Singapore, Singapore; ^3^Department of Rehabilitative Services, Changi General Hospital, Singapore, Singapore

**Keywords:** stroke, rehabilitation, robotics, robot-assisted therapy, feasibility, upper limb

## Abstract

**Objective:**

To assess the feasibility and safety of conducting robot-mediated impairment training (RMIT) and robot-mediated task-specific training (RMTT). The device deployed is the Optimo Regen (OR^®^), capable of delivering both impairment-oriented training and task-specific training.

**Methods:**

This was a single-centre, randomized, single-blinded, two-arm, parallel group, controlled trial. Patients fulfilling criteria were randomized into either the RMIT or RMIT + RMTT group and provided with 20 h of robotic therapy on top of standard care.

**Results:**

A total of 4 patients were recruited, with 2 patients receiving treatment in each arm. The study was feasible, with a 66.7% enrolment rate, 75% completion rate, and 100% attendance for each intervention session. We achieved a 90% satisfaction rate with no serious adverse effects. All patients had improvement of motor power, Fugl-Meyer Assessment-Upper Extremity (FMA-UE), Functional Independence Measure (FIM), Hospital Anxiety and Depression Scale (HADS), and quality of life scores at 1 month. FIM continued to improve at 3 months post-commencement of intervention. There was relative ease of use of the device.

**Conclusion:**

This trial is feasible. A full-scale study is warranted, to compare RMIT against RMTT, which is a novel application.

## Introduction

Stroke is among the top 10 causes of hospitalization in Singapore (1). Upper limb impairments are common after stroke (2) and may result in loss of function, including self-care activities. A Cochrane overview of systematic reviews suggests that arm function can be improved by providing at least 20 h of additional repetitive task training to patients (3). Intensity of therapy is thus important for post-stroke recovery. However, providing sufficient therapy remains a challenge due to various reasons (4), including manpower shortages. Robotic-mediated rehabilitation is an innovative exercise-based therapy using robotic devices that enables the implementation of highly repetitive, intensive, adaptive, and quantifiable physical training.

According to a study on the cost of hospital care, the bulk of the hospitalization cost went to ward charges (38.2%) with much less coming from therapy (7.3%) (5). It thus makes sense to increase the intensity of rehabilitation so that patients may recover faster and be discharged earlier. Making therapy more available in the outpatient settings where wait times are currently long would also be advantageous. In our study, patients started on robotic therapy when they were undergoing rehabilitation as inpatients and this would continue in the outpatient setting when they were discharged.

The RATULS trial (6) showed that neither robot-assisted training using the MIT-Manus robotic gym, nor an enhanced upper limb therapy (EULT) program based on repetitive functional task practice improved upper limb function after stroke, as compared to usual care, for patients with moderate-to-severe upper limb functional limitations. It was suggested that further research was needed to find ways to translate the improvements in upper limb impairments seen with robot-assisted therapy into upper limb function and their activities of daily living (ADLs).

In a systematic review and meta-analysis on the effects of robot-assisted therapy on the upper limb, it was found that although there were improvements in strength, this was not translated to improvements in ADLs (7). Additional transition to task training (facilitated by therapists) had been added to robot-mediated impairment training (RMIT) in various studies (8, 9). In a study by Hung et al. (9), robot-assisted therapy combined with occupational therapist (OT)-facilitated task specific training was found to be superior to robot-assisted therapy combined with OT-facilitated impairment-oriented training. Task-specific training consists of repetitively practicing functional tasks such as combing of hair, picking up a cup and bringing it to the mouth and so on. Impairment-oriented therapy emphasizes remediation of motor deficits with a focus on single joint movements at a time.

Although there have been publications in the engineering literature regarding different robots and their nomenclature, and envisioning how they can be deployed in therapy (10), there has been a paucity of publications on how these devices have been applied on patients, especially in performing RMTT and its functional outcomes in medical journals. A study was reported that investigated the REHAROB therapeutic system on performing RMTT. REHAROB is a robotic device used to assist patients living with chronic stroke in performing 5 ADLs and showed that patients had significant improvements on the Fugl-Meyer Assessment-Upper Extremity (FMA-UE), Action Research Arm Test (ARAT) and Functional Independence Measure (FIM) (11).

This present study aimed to determine the feasibility of the application of both RMIT and RMTT utilizing the robotic device—Optimo Regen (OR^®^). The OR^®^ is a robotic device that offers seven degrees of freedom, with the ability to provide assistance or resistance and is able to store the memory of each movement that every patient has to make in robotic therapy. From a review of the prevalent literature, there has been no study on the comparison of RMTT + RMIT against RMIT alone. A search for RMTT only yielded the REHAROB study (11), but the robot only administered RMTT and not RMIT. The preliminary effects of the intervention on physical function and quality of life would be studied.

## Methods

The study was approved by the SingHealth Centralised Institutional Review Board (CIRB 2023/2028). Written consent was obtained from all participants. This trial protocol was declared *a priori* on ClinicalTrials.gov (Identifier NCT05729633, protocol v1 dated 28th February 2023).

The hypothesis was that RMIT and RMTT conducted through the OR^®^ is safe and feasible. The objectives were to assess the feasibility and safety of conducting RMIT and RMTT through the OR^®^.

This study was a single-centre, block randomized, single-blind, two-arm, parallel-group, controlled trial. The trial protocol was developed in accordance with SPIRIT recommendations (12). The feasibility study was carried out without any external funding. The OR^®^ was loaned from HERE Life Science Pte Ltd. (Singapore), with technical support through the device inventor (Roboligent, Inc., Round Rock, TX).

Patients transferred into the inpatient rehabilitation unit at our acute general hospital with the diagnosis of stroke would be screened for suitability to participate in this study. These patients were transferred from the hospital’s acute stroke unit or medical general wards. A total of 4 patients (2 in each arm) were recruited.

### Participants

**Figure 1:**
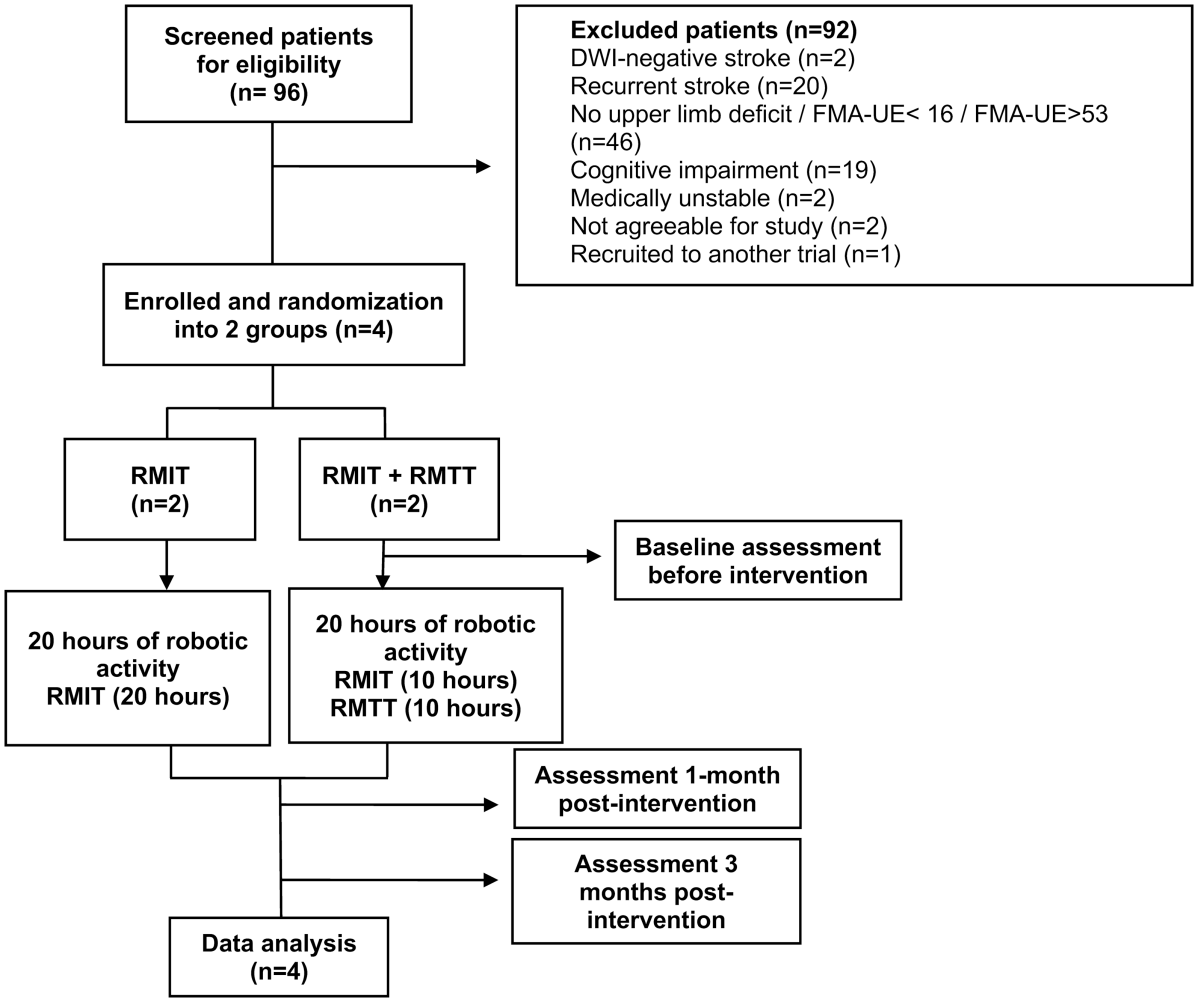
Study workflow.

All stroke patients transferred into the unit from 1st June 2023 were screened, until the 4th and final patient had been recruited. Inclusion criteria were: (1) diagnosis of stroke as evidenced by CT/MRI findings (ischaemic or haemorrhagic); (2) first-ever stroke; (3) upper limb weakness and an FMA-UE score of 16–53 (severe to moderate: 16–34; moderate to mild: 35–53) (13, 14); (4) cognitively-intact and able to follow instructions with MMSE ≥21 (5) medically stable to participate; (6) Consent given; and (7) Age 21 and above (Figure 1).

Exclusion criteria were: (1) fractures or other musculoskeletal issues that render the use of the robotic device unsuitable; (2) involvement in another concurrent upper limb study; (3) wounds that do not allow donning of the device; (4) severe spasticity; (5) severe osteoporosis; (6) infectious diseases that require the patient to be isolated in a single room e.g., airborne infections.

### Randomization

Patients were randomly assigned to 1 of the 2 groups in a 1:1 allocation ratio via REDCAP in sequence block with varying block sizes. This randomization sequence was created by the statistician who was not involved in study consent, using the STATA module “RALLOC.” To prevent subversion of the allocation sequence, study team members who enrolled patients to the trial were distinct and kept separate from the study team member that performed group assignment. Outcome assessment was performed by a study team member who was blinded to the group allocation. Unblinding was only allowed for emergencies such as life-saving situations, and at the study’s completion.

### Intervention

Patients allocated into Group 1 underwent an hour of robot-mediated therapy daily, for 5 days a week while staying inpatient, comprising 10 sessions of RMIT followed by 10 sessions of RMTT (20 h total). If the patient were to be discharged while in-flight, they would return to the hospital as an outpatient to complete the balance of the 20 sessions of robot-mediated therapy (RMT). Conventional occupational therapy was also provided. All robotic interventions were to be completed within 3 months of stroke onset.

Patients allocated to Group 2 underwent an hour of robot-mediated therapy daily, for 5 days a week while staying inpatient, comprising 20 sessions of RMIT (20 h total). If the patient were to be discharged while in-flight, they would return to the hospital as an outpatient to complete the balance of the 20 sessions of robot-mediated therapy (RMT). Conventional occupational therapy was provided. All robotic interventions were to be completed within 3 months of stroke onset.

RMIT focused on the following movements: (1) diagonal movement; (2) shoulder abduction; (3) shoulder adduction; (4) shoulder flexion; (5) shoulder extension; (6) elbow flexion; (7) elbow extension (Supplementary Video S1). RMTT focused on the following activities: (1) picking up a cup/glass by the side and drinking; (2) brushing hair (Supplementary Video S2); (3) cleaning unaffected upper limb (hand to arm); (4) wiping table; (5) wiping a wall; (6) sliding a card on the table to a designated location; (7) clipping a clothe peg.

### The Optimo Regen robot

The OR^®^ uses force-control technology as opposed to traditional position/velocity-control robots, to mimic natural kinematic movements. Different from other robots that restrict patients to constrained movement axes, the OR^®^ uses proprietary precision torque control actuators installed in a 7-jointed arm to deliver not only highly precise low-impedance force control, but also a high load capacity over a large force bandwidth. This allows the robot to be sensitive to small improvements in a patient’s muscle power, and yet be able to support patients’ escalating therapy needs up to higher-resistance strength training while facilitating natural limb movements. The robot’s other features include a teach-and-follow mode for repetitive movements, configurability for arm or leg use, and a range of hardware-dependent and independent safety stops. The OR^®^ is capable of both delivering RMIT as well as RMTT. It can provide zero, partial, or full assistance to the patient to complete the movement or task. Its teach-and-follow mode allows a movement to be performed by the therapist, with the device then “replaying” the movement at either zero, partial, or full assistance, for the patient.

For each movement, the patient would be guided passively through the movement for it to be recorded. Thereafter, the amount of assistance, or weight support was programmed for each specific patient. This would be progressively reduced as the patient got stronger. If the patient was not able to complete the whole range of motion within a certain time, the device would give a subtle nudge, then bring the patient through the movement if he or she were unable to progress.

### Outcome measures

This was a single-blinded study, with a masked assessor collecting routine outcome measures at the following time points: at baseline, 1 month, and 3 months post-commencement of the intervention at the rehabilitation unit. We maintained separation between study team members that assessed outcomes, and the one that performed group assignment. At the start of every assessment, patients would be reminded not to reveal their treatment assignment by referring to elements of the treatment that were unique to their treatment group. The main outcome measure was feasibility measured in terms of enrolment rate, completion rate, compliance rate, patient satisfaction, and the presence of adverse effects, including fatigue, pain, and injuries. Secondary outcome measures include FMA-UE, FMA-UA, FMA-W/H, FAT, FIM, muscle power by MRC, MAS, EQ-5D-5L, and HADS,

### Feasibility outcomes

Feasibility was measured in terms of enrolment rate, completion rate, compliance rate, patient satisfaction, and the presence of adverse effects, including fatigue, pain and injuries. The enrolment rate was defined as the number of participants successfully enrolled out of the total number of participants deemed eligible. The completion rate is the percentage of participants who completed 20 h of intervention and had their outcome measures collected at the baseline, 1 month and 3 months post-commencement of intervention. The compliance rate is defined as the patient turning up for each robotic intervention session. The patient satisfaction survey was administered at the 1-month and 3-months post commencement of intervention by the masked assessor. The presence of adverse effects was recorded at the 1-month and 3-month post-intervention timepoints by the masked assessor, as well as direct questioning prior to and during each intervention session by the study team.

### UE impairment and function

Upper extremity impairment was measured with the Fugl-Meyer Assessment for Upper Extremity (FMA-UE) motor recovery (15). This is a frequently used outcome scale to measure post stroke motor recovery of the upper limb. Secondary outcome measures include the Fugl-Meyer Assessment-Upper Arm (FMA-UA) and Fugl-Meyer Assessment-Wrist/Hand (FMA-W/H) which are subsets of the FMA-UE. The Frenchay Arm Test (FAT) is a measure of upper extremity proximal motor control and dexterity during ADL performance (16). Other measures of upper limb function include muscle power by the Medical Research Council (MRC), and Modified Ashworth Scale (MAS) which is a spasticity scale.

### Other functional outcome measures

The Functional Independence Measure (FIM) assesses 6 areas of function and was developed to offer a uniform system of measurement for disability (17). It, as well as its subscores, offer a detailed and holistic gestalt of the sum disability of patients in ADLs such as mobility and toileting.

### Health-related quality-of-life

Health-related quality-of-life scales include EQ-5D-5L which is a quality-of-life questionnaire (18), and the Hospital Anxiety and Depression Scale (HADS) (19) which is a measurement of mood. The EQ-5D-5L is an instrument that evaluates the quality of life, considering five dimensions that include mobility, self-care, usual activities, pain and discomfort, and anxiety and depression. These are rated on a 5-point Likert-like scale, and the higher the score on the EQ-5D-5L, the better the outcome. The HADS questionnaire includes seven items each for depression and anxiety subscales. Each individual item’s score ranges from zero to three, with three denoting the highest level of anxiety or depression. A total subscale score of 8 or more denotes possible anxiety or depression.

### Data management

Patient demographics and outcome measures would be de-identified and collated in a password-protected cloud-based platform (REDCap). All hardcopy forms and electronic records pertaining to the participants’ data would be retained for a minimum of 5 years before destruction.

### Data analysis

We reasoned that a sample size of 4 participants (2 in each arm) would give us an appropriate trade-off between rigor and pragmatism. First, as the primary objective was to assess the feasibility of a larger, more definitive trial, a smaller sample allowed for a more nuanced, case-by-case analysis of each participant’s experience and response to the intervention. Second, resource and logistical constraints necessitated a smaller sample size. This study was not powered to detect differences in clinical outcomes between the two groups.

Reporting of trial results was in accordance with the CONSORT 2010 extension to randomized pilot and feasibility trials guidelines (20). Participants’ baseline demographic and clinical characteristics were reported in detail for each participant. Similarly, a case-level analysis was performed for the feasibility and clinical outcomes across time. The change in the outcome at 1 and 3 months was calculated by subtracting the baseline value of the outcome from its respective values at 1 and 3 months. The satisfaction level (in %) was calculated by dividing the total satisfaction score across eight 5-point items by the maximum score of 40, multiplied by 100. No inferential statistics were reported in this feasibility trial. All analyses were conducted using Stata 18 (College Station, TX: StataCorp LLC).

## Results

Ninety-six patients with stroke were screened for eligibility between the period of 1 June 2023 to 24 August 2023, of which 6 patients met the inclusion criteria (Figure 1). One patient declined as she was a foreign national who would not be able to stay in Singapore until the end of the study. Another declined due to inability to return to the hospital for outpatient RMT from a lack of caregiver support. Four patients were subsequently recruited and randomized into both arms. The patient demographic and clinical characteristics are presented in Table 1. These patients started training with the robotic arm between day 7 and day 16 of their stroke onsets.

**Table 1:** Patients’ baseline characteristics.

	ID1	ID2	ID3	ID4
Age	42	50	54	66
Sex	Male	Male	Male	Male
Race	Malay	Chinese	Chinese	Others
Co-morbidities	DM, HTN	Nil	Nil	Nil
Hand dominance	Right	Right	Right	Right
Pre-morbidly working	Yes	Yes	Yes	Yes
Presence of caregiver	No	No	No	No
Stroke type	Infarct	Haemorrhage	Infarct	Infarct
First ever stroke	Yes	Yes	Yes	Yes
Affected side	Right	Right	Right	Right
Days from stroke to enrolment	12	16	7	12
Baseline function status at time of randomisation				
MMSE	25	21	25	29
NIHSS	8	NA	10	7
Charlson comorbidity index (CCI)	1	1	1	2

### Feasibility

All patients completed baseline, 1-month and 3-month functional outcome assessments except for patient #4 who declined to answer the health-related quality-of-life outcome measures at the 3-month time point. All participants completed the full 20 h of robotic therapy (Table 2). Most of the interventions were completed within the first month of commencement of robotic therapy (Supplementary Table S1).

**Table 2:** Feasibility outcome and quality of life.

	ID1			ID2			ID3			ID4		
	Base	1 month	3 months	Base	1 month	3 month	Base	1 month	3 months	Base	1 month	3 months
	RMIT			RMIT + RMTT			RMIT			RMIT + RMTT		
Compliance												
Completed 20 h of RT (yes/no)			Yes			Yes			Yes			Yes
Compliance with the regimen	Yes	Yes	Yes	Yes	Yes	Yes	Yes	Yes	Yes	Yes	Yes	Yes
Quality of life												
EQ-5D (range: 0–100)	100	80	70	55	80	80	40	75	80	50	70	Missing
HADS-A (range: 0–42)	4	3	1	2	1	1	2	6	3	2	0	Missing
HADS-D (range: 0–42)	4	2	2	8	3	8	9	4	1	7	4	Missing
Satisfaction survey [range: 8–40; *n* (%)]		40 (100)	40 (100)		37 (92.5)	36 (90)		39 (97.5)	40 (100)		36 (90)	Missing
Adverse events												
Fatigue	Nil	Nil	Nil	Nil	Nil	Nil	Nil	Nil	Nil	Nil	Nil	Nil
Injuries	Nil	Nil	Nil	Nil	Nil	Nil	Nil	Nil	Nil	Nil	Nil	Nil
Pain/discomfort	Nil	Nil	Nil	Nil	Nil	Present	Nil	Nil	Nil	Nil	Nil	Nil
Spasticity	Nil	Nil	Yes	Nil	Nil	Nil	Nil	Nil	Nil	Nil	Nil	Yes
Others	Blurry vision	Nil	Nil	Nil	Nil	Nil	Nil	Nil	Nil	Nil	Nil	Nil

The intensity of application was an hour daily, 5 days a week while all patients were staying for inpatient rehabilitation. Upon discharge, the intensity of application ranged from 3 to 5 times a week, for an hour each session.

The enrolment rate was 66.7%. 100% of the patients fully completed the intervention. Compliance was 100% as each patient turned up for each scheduled robotic session. Completion rate was 75% as patient #4 declined to complete the quality-of-life outcomes. On the patient satisfaction survey, at least 90% satisfaction was achieved across all domains, such as ease-of-use, enjoyment, and intention to tell other patients about the robotic device.

No serious adverse effects were reported relating to the intervention. One patient complained of blurred vision during his stay, which was found to be unrelated to the intervention. At the 3-month follow-up, 2 patients were found to have slight worsening of spasticity. and one of the patients was found to have developed new medical complications of acute kidney injury and post-stroke depression. There were no reports of fatigue, pain, or injuries during the use of the robotic device.

### Physical function

**Figure 2:**
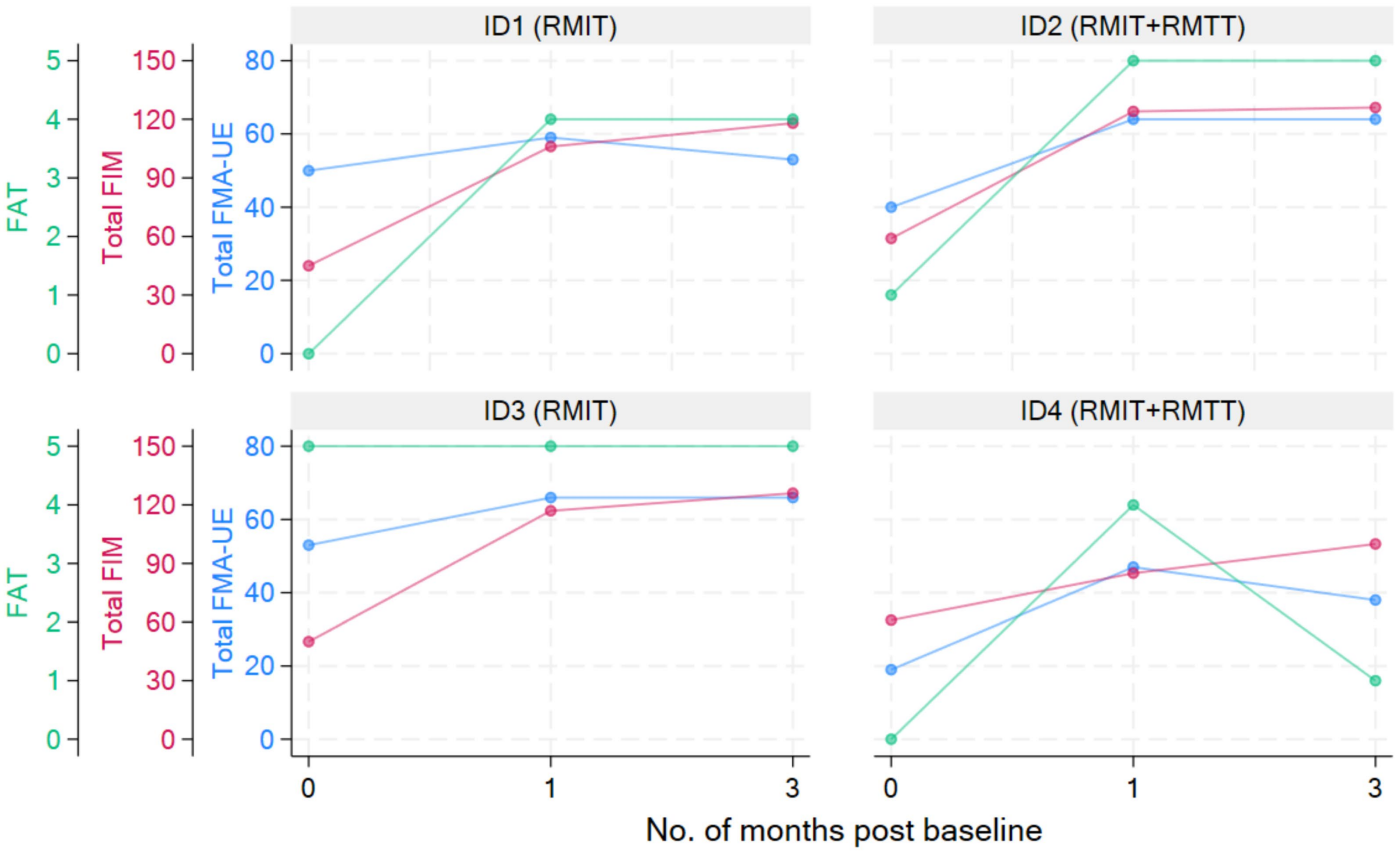
Graphical presentation of clinical outcomes over time.

All patients were assessed to have improvement in strength as measured by the MRC. All patients had improvements in FMA-UE. Three patients had improvements in FAT. All patients improved in their FIM (Figure 2 and Tables 3, 4).

**Table 3:** Physical outcome measures: FMA and FIM.

	ID1			ID2			ID3			ID4		
	Base	1 month	3 months	Base	1 month	3 months	Base	1 month	3 months	Base	1 month	3 months
	RMIT			RMIT + RMTT			RMIT			RMIT + RMTT		
FMA-UE												
A. FMA-UA	31	36	32	27	36	36	33	36	36	9	26	19
B. FMA-Hand/Wrist	19	22	21	13	22	22	19	24	24	10	20	19
C. FMA-Coordination/Speed	0	1	2	0	6	6	1	6	6	0	1	0
D. FMA-UE	50	59	53	40	64	64	53	66	66	19	47	38
FIM												
Motor (ADLs & mobility)	25	70	87	40	91	91	18	86	91	27	50	65
Cognitive	20	35	34	19	33	35	32	34	35	35	35	35
Total FIM (range: 18–126)	45	106	118	59	124	126	50	117	126	61	85	100
FAT (range: 0–5)	0	4	4	1	5	5	5	5	5	0	4	1

**Table 4:** Physical outcome measures: MMT and MAS.

	ID1			ID2			ID3			ID4		
	Base	1 month	3 months	Base	1 month	3 months	Base	1 month	3 months	Base	1 month	3 months
	RMIT			RMIT + RMTT			RMIT			RMIT + RMTT		
MMT (range: 0–5)												
Shoulder flexion	3	4	5	3	5	5	5	5	5	1	3	1
Shoulder extension	3	4	5	3	5	5	5	5	5	1	3	0
Shoulder abduction	3	5	5	3	5	4	4	5	5	2	4	5
Shoulder adduction	4	5	5	3	5	5	5	5	5	4	4	3
Shoulder internal rotation	3	4	5	3	5	5	5	5	5	3	4	4
Shoulder external rotation	3	4	5	3	5	5	4	5	5	1	3	2
Elbow flexion	4	5	5	3	5	5	5	5	5	2	4	4
Elbow extension	4	5	5	3	5	5	5	5	5	4	4	3
Pronation	4	5	5	3	5	5	5	5	5	4	4	4
Supination	4	5	5	3	5	5	5	5	5	2	4	3
Wrist flexion	5	5	5	3	5	5	5	5	5	4	4	3
Wrist extension	4	5	5	3	5	5	5	5	5	4	4	3
Finger flexion	4	5	5	3	5	5	5	5	5	4	4	5
Finger extension	5	5	4	3	5	5	5	5	5	3	4	3
Thumb flexion	4	5	5	3	5	5	5	5	5	4	4	4
MAS (range: 0–4)												
Shoulder flexor	0	0	0	0	0	0	0	0	0	0	0	0
Shoulder extensor	0	0	0	0	0	0	0	0	0	0	0	1
Shoulder abductor	0	0	0	0	0	0	0	0	0	0	0	0
Shoulder adductor	0	0	0	0	0	0	0	0	0	0	1	0
Shoulder internal rotator	0	0	0	0	0	0	0	0	0	1	0	2
Shoulder external rotator	0	0	0	0	0	0	0	0	0	0	0	0
Elbow flexor	0	0	1	0	0	0	0	0	0	0	1	1
Elbow extensor	0	0	1	0	0	0	0	0	0	2	1	2
Pronator	0	0	0	0	0	0	1	0	0	0	0	0
Supinator	0	0	0	0	0	0	0	0	0	0	0	0
Wrist flexors	0	0	0	0	0	0	0	0	0	0	0	0
Wrist extensors	0	0	0	0	0	0	0	0	0	0	0	0
Finger flexors	0	0	0	0	0	0	0	0	0	0	0	0
Finger extensors	0	0	0	0	0	0	0	0	0	0	0	0
Thumb flexors	0	0	0	0	0	0	0	0	0	0	0	0

### Quality of life

There was improvement in the EQ-5D-5L for 2 patients and a deterioration in 1 patient at the 1-month and 3-month periods as compared to the baseline. Three patients improved in their HADS scores at 1 month and 3 months compared to baseline, whereas 1 patient experienced deterioration (Table 2).

### Ease of use of device

It took an hour to train the research team members to operate the device as well as to program the exercises. Following randomization and prior to the start of each patient’s training series, it required approximately half an hour to program and record their exercises into the device’s memory. The RMIT and RMTT exercises are standard, but the differences in limb size required that each movement be calibrated. Donning and doffing the device took approximately 3 to 5 min each. Once training began, patients could be supervised by a therapist assistant. The device’s assistance level for each patient was reviewed every other session, and the amount of assistance was reduced when they had made significant improvements or began to find the training less effortful.

### Technical issues

There were 2 instances when the device overheated, and these were resolved with switching on the air-conditioning (Singapore’s average daytime temperature is 31°C). There were 4 instances at the start of the study when the arm froze in place and the intervention session was not able to continue. This had to be resolved by the supplier’s technical support staff. One such instance occurred when the support staff was overseas, and the technical issue was resolved over a video call. The total loss of time to the intervention schedule due to this was 2 days.

## Discussion

Studies have yet to prove the efficacy of robot-assisted training over usual care (3). There are several challenges in the interpretation of these studies. The duration of robotic therapy applied has been extremely heterogeneous, ranging from 2 to 12 weeks, with total therapy times of 0.5 to 90 h, and repetition counts running from 50 to 2,700 per session (7). Secondly, there are various robotic devices ranging from exoskeletons to end-effectors, devices that train the shoulder and elbow to the hand and fingers (7). Many robotic devices often move in specific planes or assist the upper limb to make isolated joint movements. Patients trained on these would then need to re-learn how to combine these isolated movements into a smooth three-dimensional, multi-joint movement when performing an ADL. Thirdly, studies are often not designed to provide complementary task-specific training in addition to robotic therapy (6, 9). Fourthly, the acuity of the stroke is related to the impact of the intervention on neurorecovery. For example, in the RATULS trial, the recruited patients ranged from 1 week to 5 years post-stroke (median of 240 days). A paucity of literature surrounds the efficacy of robotic therapy within the first 3 months post-stroke (7), during the period where neuroplasticity is at its highest (21), and training effects would be expected to be the most marked.

Our study was designed to investigate the feasibility of applying intensive robotic therapy within the first 3 months of stroke. Patients were recruited during the acute phase of stroke. The novelty is in being able to repeatedly train the patient in performing task-specific activities leveraging on robotic therapy. Although head-to-head comparisons between robotic therapy and conventional therapy suggested non-inferiority in functional gains, these described robots typically carried out training in 2 dimensions, and further translation of such robotic devices to functional tasks remained unknown.

In our study, robotic therapy was accompanied by conventional occupational therapy while the patients were admitted. Undergoing robotic therapy sessions did not compromise the patients’ ability to participate in their conventional sessions, and these complementary sessions allowed our patients to have a higher cumulative dose of upper limb repetitions than their counterparts would have had. Our study had shown that it is feasible to apply 20 h of robotic therapy within the first month of a stroke, let alone 3 months as was initially envisaged.

No specific hand training was targeted together with the robotic therapy, as the hand component of the OR^®^ has yet to be developed. Hand training took place according to the patient’s needs at the conventional occupational therapy sessions. All of our patients had a minimal hand grip strength of at least 3. Patients with reduced hand grip strength may benefit from the use of universal cuffs with or without wrist support and Velcro Straps during robotic training.

Most improvements occurred within the first month of intervention, and the effects were sustained at 3 months post-intervention except for patients #1 and #4, who were randomized to different intervention arms. The common situation that both faced was slow commencement of community-based therapy in the outpatient rehabilitation centers (delay of up to 6 weeks post-discharge), leading to disuse-related deconditioning and a deterioration of the FMA, with patient #4 also experiencing deteriorations in his manual muscle testing. All the patients experienced a minimal clinically important difference (MCID) (22) in their improvement in the FMA-UE at both the 1-month and 3-month timepoints except for patient #1 who deteriorated at the 3-month timepoint. The MCID for FMA-UE ranged from 4 to 12.4 in various studies (22). The increase in spasticity in both of these patients at the 3-month timepoint was likely related to the lack of therapy post discharge. In the latter’s case, these were related to his post-stroke medical complications and not due to the use of the robotic device. This suggests that a seamless transition to outpatient rehabilitation is of paramount importance to sustaining rehabilitation gains. Although physical scores peaked at 1-month post-intervention in patients #1–#3, their FIM scores continued to rise at the 3-month mark, illustrating how neurological and functional arm recovery is required for subsequent translation into the performance of ADLs. All patients exceeded the MCID (improvement of 22 points) (22) for total FIM improvement at both 1 and 3 months post intervention.

Rehabilitation is manpower-intensive and costly. With global manpower shortages, the intensity of and frequency of stroke rehabilitation is often unable to meet the daily durations recommended in international guidelines (23). This is aligned with local research suggesting that substitution of a portion of conventional therapy with robotic therapy may reduce workforce demands (24). Robotic therapy also allows us a novel means to bridge the inpatient-outpatient interruption of therapy provision, through alleviation of the manpower requirements that would necessitate a neurological occupational therapist or physiotherapist experienced enough to care for patients with acute-to-subacute stroke. This could be envisioned through allowing patients to continue accessing robotic therapy using their recorded settings, for up to 3 months while awaiting commencement of their community-based therapy.

The utilization of robotics for both RMIT and RMTT may be a further step forward in either the reduction of manpower demands or in increasing training dosage, optimizing functional recovery so as to alleviate the burden of care. A robotic group therapy setting could be designed for both inpatients and outpatients, that will allow a therapist to simultaneously monitor several patients at the same time, further optimizing manpower efficiency. A trained therapist assistant could be engaged to supervise experienced patients once *ad-hoc* calibrations and assistance level adjustments have been made.

The main challenge of deploying a high-end smart robot in clinical practice would be its construction and licensing costs. Financial cost-savings analyses that assess the feasibility of purchasing such a platform should however also take into account clinician manpower savings, relief of caregiver burden, as well as the potential for patients with good post-stroke recovery to return to competitive employment and become economically productive.

### Study limitations

This is a feasibility study, and its weakness is that of a small sample size. We estimated the sample size in accordance with DELTA2 guidance (25). In a future RCT to assess efficacy, the primary endpoint would be FMA-UE score at 1- and 3-months post baseline. The reported minimal clinically important difference (MCID) for FMA-UE scores in 12 convalescent stroke patients with moderate to severe hemiparesis from 3 hospitals in Japan at 6-weeks post baseline was 12.4 (14). In a two-arm, parallel-group, superiority trial with 1:1 allocation, to detect a difference in FMA-UE score of 12.4 with a two-sided 5% significance and 80% power, assuming an FMA-UE score standard deviation of 19.32 and a drop-out rate of 15%, the minimum sample size required would be 48 patients per arm (96 total). We estimate a sample size of 96 would be needed to draw robust conclusions between the outcomes of RMTT and RMIT. Another limitation that we recognized was the stringent inclusion criteria due to its nature of being a research project and an RCT. We noticed that many other patients could have benefited from robotic therapy and subsequent work should refrain from such restrictive criteria.

## Conclusion

This trial is feasible. A full-scale study is warranted, to compare RMIT against RMIT and RMTT which is a novel application. The robotic device’s efficacy in improving ADLs should be explored. Future studies should be designed such that they will be clinically applicable, with logistical considerations to allow a greater number of patients to be simultaneously scheduled, as well as transport cost defrayment for those who need to travel for their outpatient sessions.

## Data Availability

The original contributions presented in the study are included in the article/Supplementary material, further inquiries can be directed to the corresponding author.

## Supplementary material

SUPPLEMENTARY TABLE S1 Number of OR sessions and hours of OR sessions attended in 1 month.

SUPPLEMENTARY VIDEO S1 RMIT elbow flexion extension.

SUPPLEMENTARY VIDEO S2 RMTT hair combing.

## References

[1] Venketasubramanian N Chen CL. Burden of stroke in Singapore. Int J Stroke. (2008) 3:51–4. doi: 10.1111/j.1747-4949.2008.00181.x 18705915

[2] Langhorne P Coupar F Pollock A. Motor recovery after stroke: a systematic review. Lancet Neurol. (2009) 8:741–54. doi: 10.1016/S1474-4422(09)70150-4 19608100

[3] Pollock A Farmer SE Brady MC Langhorne P Mead GE Mehrholz J. Interventions for improving upper limb function after stroke. Cochrane Database Syst Rev. (2014) 2014:CD010820. doi: 10.1002/14651858.CD010820.pub2, PMID: 25387001 PMC6469541

[4] Clarke DJ Burton LJ Tyson SF Rodgers H Drummond A Palmer R. Why do stroke survivors not receive recommended amounts of active therapy? Findings from the ReAcT study, a mixed-methods case-study evaluation in eight stroke units. Clin Rehabil. (2018) 32:1119–32. doi: 10.1177/0269215518765329, PMID: 29582712 PMC6068965

[5] Venketasubramanian N Yin A. Hospital costs for stroke care in Singapore. Cerebrovasc Dis. (2000) 10:320–6. doi: 10.1159/000016077 10878439

[6] Rodgers H Bosomworth H Krebs HI van Wijck F Howel D Wilson N. Robot assisted training for the upper limb after stroke (RATULS): a mulitcentre randomised controlled trial. Lancet. (2019) 394:51–62. doi: 10.1016/S0140-6736(19)31055-4, PMID: 31128926 PMC6620612

[7] Veerbeek JM Langbroek-Amersfoort AC van Wegen EE Meskers CG Kwakkel G. Effects of robot-assisted therapy for the upper limb after stroke. Neurorehabil Neural Repair. (2017) 31:107–21. doi: 10.1177/1545968316666957 27597165

[8] Conroy SS Wittenberg GF Krebs HI Zhan M Bever CT Whitall J. Robot-assisted arm training in chronic stroke: addition of transition-to-task practice. Neurorehabil Neural Repair. (2019) 33:751–61. doi: 10.1177/1545968319862558, PMID: 31328671

[9] Hung CS Hsieh YW Wu CY Lin YT Lin KC Chen CL. The effects of combination of robot-assisted therapy with task-specific or impairment-oriented training on motor function and quality of life in chronic stroke. PM R. (2016) 8:721–9. doi: 10.1016/j.pmrj.2016.01.008, PMID: 26805909

[10] Dalla Gasperina S Roveda L Pedrocchi A Braghin F Gandolla M. Review on patient-cooperative control strategies for upper-limb rehabilitation exoskeletons. Front Robot AI. (2021) 8:745018. doi: 10.3389/frobt.2021.745018, PMID: 34950707 PMC8688994

[11] Peter O Tavaszi I Toth A Fazekas G. Exercising daily living activities in robot-mediated therapy. J Phys Ther Sci. (2017) 29:854–8. doi: 10.1589/jpts.29.854, PMID: 28603359 PMC5462686

[12] Chan AW Tetzlaff JM Altman DG Laupacis A Gøtzsche PC Krleža-Jerić K. SPIRIT 2013 statement: defining standard protocol items for clinical trials. Ann Intern Med. (2013) 158:200–7. doi: 10.7326/0003-4819-158-3-201302050-00583, PMID: 23295957 PMC5114123

[13] Woytowicz EJ Rietschel JC Goodman RN Conroy SS Sorkin JD Whitall J. Determining levels of upper extremity movement impairment by applying cluster analysis to upper extremity Fugl-Meyer assessment in chronic stroke. Arch Phys Med Rehabil. (2017) 98:456–62. doi: 10.1016/j.apmr.2016.06.023, PMID: 27519928 PMC5299057

[14] Hiragami S Inque Y Harada K. Minimal clinically important difference for the Fugl-Meyer assessment of the upper extremity in convalescent stroke patients with moderate to severe hemiparesis. J Phys Ther Sci. (2019) 31:917–21. doi: 10.1589/jpts.31.917, PMID: 31871377 PMC6879402

[15] Fugl-Meyer AR Jääskö L Leyman I Olsson S Steglind S. The post-stroke hemiplegic patient. 1. A method for evaluation of physical performance. Scand J Rehabil Med. (1975) 7:13–31. doi: 10.2340/1650197771331, PMID: 1135616

[16] Laclergue Z Ghédira M Gault-Colas C Billy L Gracies JM Baude M. Relaibility of the modified Frenchay scale for the assessment of upper limb function in adults with hemiparesis. Arch Phys Med Rehabil. (2023) 104:1596–605. doi: 10.1016/j.apmr.2023.04.003, PMID: 37121532

[17] Galloway RV Granger CV Karmarkar AM Graham JE Deutsch A Niewczyk P. The uniform data system for medical rehabilitation report of patients with debility discharged from inpatient rehabilitation programs 2000–2010. Am J Phys Med Rehabil. (2013) 92:14–27. doi: 10.1097/PHM.0b013e31827441bc, PMID: 23117268 PMC3652310

[18] Rabin R de Charro F. EQ-5D: a measure of health status from the EuroQol Group. Ann Med. (2001) 33:623–34. doi: 10.3109/07853890109002087, PMID: 11491192

[19] Edelstein BA Drozdick LW Ciliberti CM. Hospital Anxiety and Depression Scale In: Handbook of assessment in clinical gerontology. 2nd ed. Cambridge, MA: Elsevier Academic Press. (2010)

[20] Eldridge SM Chan CL Campbell MJ Bond CM Hopewell S Thabane L. CONSORT 2010 statement: extension to randomised pilot and feasibility trials. BMJ. (2016) 355:i5239. doi: 10.1136/bmj.i5239 27777223 PMC5076380

[21] Murphy T Corbett D. Plasticity during stroke recovery: from synapse to behaviour. Nat Rev Neurosci. (2009) 10:861–72. doi: 10.1038/nrn2735 19888284

[22] Mishra B Sudheer P Agarwal A Nilima N Srivastava MVP Vishnu VY. Minimal clinically important difference of scales reported in stroke trials: a review. Brain Sci. (2024) 14:80. doi: 10.3390/brainsci14010080, PMID: 38248295 PMC10813687

[23] Winstein CJ Stein J Arena R Bates B Cherney LR Cramer SC. Guidelines for adult stroke rehabilitation and recovery: a guideline for healthcare professionals from the American Heart Association/American Stroke Association. Stroke. (2016) 7:e98–e169. doi: 10.1161/STR.0000000000000098 27145936

[24] Budhota A KSG C Hussain A Kager S Cherpin A Contu S. Robot assisted upper limb training post stroke: a randomized control trial using combinatory approach toward reducing workforce demands. Front Neurol. (2021) 12:622014. doi: 10.3389/fneur.2021.622014, PMID: 34149587 PMC8206540

[25] Cook JA Julious SA Sones W Hampson LV Hewitt C Berlin JA. DELTA^2^ guidance on choosing the target difference and undertaking and reporting the sample size calculation for a randomised controlled trial. Trials. (2018) 19:606. doi: 10.1186/s13063-018-2884-0 30400926 PMC6218987

