# Supplementary material for: Robot-mediated impairment-oriented and task-specific training on upper limb post stroke: feasibility and preliminary effects on physical function and quality of life
Source: Front Neurol. 2024 Oct 11;15:1415773. doi: 10.3389/fneur.2024.1415773 (PMC11505121; doi:10.3389/fneur.2024.1415773)
Supplement: SUPPLEMENTARY TABLE S1 — Number of OR sessions and hours of OR sessions attended in 1 month. [file Data_Sheet_1.PDF]

Supplementary Table 1.

Identifier	Number of OR sessions attended in 1 month	Number of hours of OR in 1 month (Hours)
**ID1**	17	16.5
**ID2**	20	20
**ID3**	20	19
**ID4**	20	20
